# Integration of Gastric Cancer RNA‐Seq Datasets Along With PPI Network Suggests That Nonhub Nodes Have the Potential to Become Biomarkers

**DOI:** 10.1002/cnr2.70126

**Published:** 2025-01-24

**Authors:** Akram Siavoshi, Mehran Piran, Ali Sharifi‐Zarchi, Fatemeh Ataellahi

**Affiliations:** ^1^ Department of Alborz Health Technology Development Center Alborz University of Medical Sciences Alborz Iran; ^2^ Department of Medical Biotechnology, Drug Design and Bioinformatics Unit, Biotechnology Research Center Pasteur Institute of Iran Tehran Iran; ^3^ Department of Computer Engineering Sharif University of Technology Tehran Iran; ^4^ Department of Biology, College of Sciences Shiraz University Shiraz Iran

**Keywords:** biomarker discovery, gastric cancer, IGFBP2, integration of RNA‐seq datasets, nonhubs, protein–protein interaction network

## Abstract

**Background:**

The breakthrough discovery of novel biomarkers with prognostic and diagnostic value enables timely medical intervention for the survival of patients diagnosed with gastric cancer (GC). Typically, in studies focused on biomarker analysis, highly connected nodes (hubs) within the protein–protein interaction network (PPIN) are proposed as potential biomarkers. However, this study revealed an unexpected finding following the clustering of network nodes. Consequently, it is essential not to overlook weakly connected nodes (nonhubs) when determining suitable biomarkers from PPIN.

**Methods and Results:**

In this study, several potential biomarkers for GC were proposed based on the findings from RNA‐sequencing (RNA‐Seq) datasets, along with differential gene expression (DGE) analysis, PPINs, and weighted gene co‐expression network analysis (WGCNA). Considering the overall survival (OS) analysis and the evaluation of expression levels alongside statistical parameters of the PPIN cluster nodes, it is plausible to suggest that THY1, CDH17, TGIF1, and AEBP1, categorized as nonhub nodes, along with ITGA5, COL1A1, FN1, and MMP2, identified as hub nodes, possess characteristics that render them applicable as biomarkers for the GC. Additionally, insulin‐like growth factor (IGF)‐binding protein‐2 (IGFBP2), classified as a nonhub node, demonstrates a significant negative correlation with both groups within the same cluster. This observation underscores the conflicting findings regarding IGFBP2 in various cancer studies and enhances the potential of this gene to serve as a biomarker.

**Conclusion:**

The findings of the current study not only identified the hubs and nonhubs that may serve as potential biomarkers for GC but also revealed a PPIN cluster that includes both hubs and nonhubs in conjunction with IGFBP2, thereby enhancing the understanding of the complex behavior associated with IGFBP2.

## 1 Background

The modern world of biology and concerned researchers believe that prolonged delays in the pathological diagnosis and prognosis of cancers result in missed treatment opportunities and impose substantial financial burdens associated with care. Exploring novel biomarkers with prognostic and diagnostic value provides timely medical intervention for survival of patients with gastric cancer (GC) [1, 2, 3].

Currently, GC is recognized as the fourth leading cause of cancer‐related fatalities globally, particularly affecting populations in East Asia. A contributing factor to the high mortality rate is that patients with GC often seek medical intervention only when the disease has progressed to an advanced stage with the tumor having metastasized to other parts of the body. Consequently, developing early and noninvasive diagnostic and prognostic biomarkers that can effectively monitor and predict cancer progression at various stages is paramount [4, 5, 6].

In numerous studies focused on cancer, RNA‐Seq has been extensively employed as a standalone technique for DGE analysis. However, a large number of differentially expressed genes (DEGs) achieve statistical significance, prompting the use of both PPIN and WGCNA as methodologies to filter these genes and associate them with disease phenotypes, cellular structures, and biological pathways. The WGCNA facilitates the identification of co‐expressed genes, while PPIN aids in clustering proteins linked to each gene, thereby contributing to identifying genes closely associated with the disease [7, 8, 9, 10].

The PPIN serves as a collective representation and association of proteins arising from biochemical processes within the cell. In this kind of network, the nodes symbolize proteins, while the edges denote the interactions between the proteins. The topological parameters of the network are established based on the connections and interactions among the nodes [11, 12].

The primary topological parameters of PPIN, such as degree (*K*), Betweenness Centrality (BC), and Closeness Centrality (CC), play a crucial role in elucidating the function of proteins (network nodes) within disease‐related signaling pathways. In this context, the degree of a node is quantified by the number of edges associated with it. The BC serves as a centrality measure within a graph, focusing on the shortest paths (SPs) and indicating the frequency with which a node appears on these paths between pairs of nodes [13]. This metric helps identify nodes that act as intermediaries connecting different sections of a graph. CC, on the other hand, provides an estimate of the speed at which information can flow from a given node to other nodes. Consequently, proteins exhibiting high values of *K* and BC are considered to be significantly interconnected with other proteins, forming clusters within the PPIN, and are commonly referred to as hub nodes. Conversely, nodes characterized by low *K* and BC, situated among hub nodes, are identified as nonhubs [14].

In numerous studies, it has been demonstrated that clustered hub nodes significantly influence the majority of activities essential for cellular survival and the progression of diseases, making them suitable candidates for evaluation through various laboratory and clinical phases before being recognized as biomarkers [15, 16].

Nonhubs are considered to have less impact on the development of biological processes, leading to the lack of the capacity to serve as clinical biomarkers. Despite the limited number of bioinformatics studies exploring the potential of nonhubs as biomarkers, this topic has garnered interest due to their significant impact on pharmacological and biological characteristics. According to these investigations, nonhubs in biological systems influence biological processes primarily through interacting with hub nodes [17, 18, 19].

Clearly, a full explanation of the interaction between PPIN nodes (hubs and nonhubs) plays a crucial role in identifying approaches that enhance exploratory processes for evaluating various biomarkers. In contrast to earlier research, this study uniquely investigates the potential of utilizing hubs and nonhubs (The less prominent nodes within the PPI network) as biomarkers in GC [20, 21, 22].

## 2 Material and Methods

### 2.1 Datasets Resources and Integration Pipeline

**FIGURE 1:**
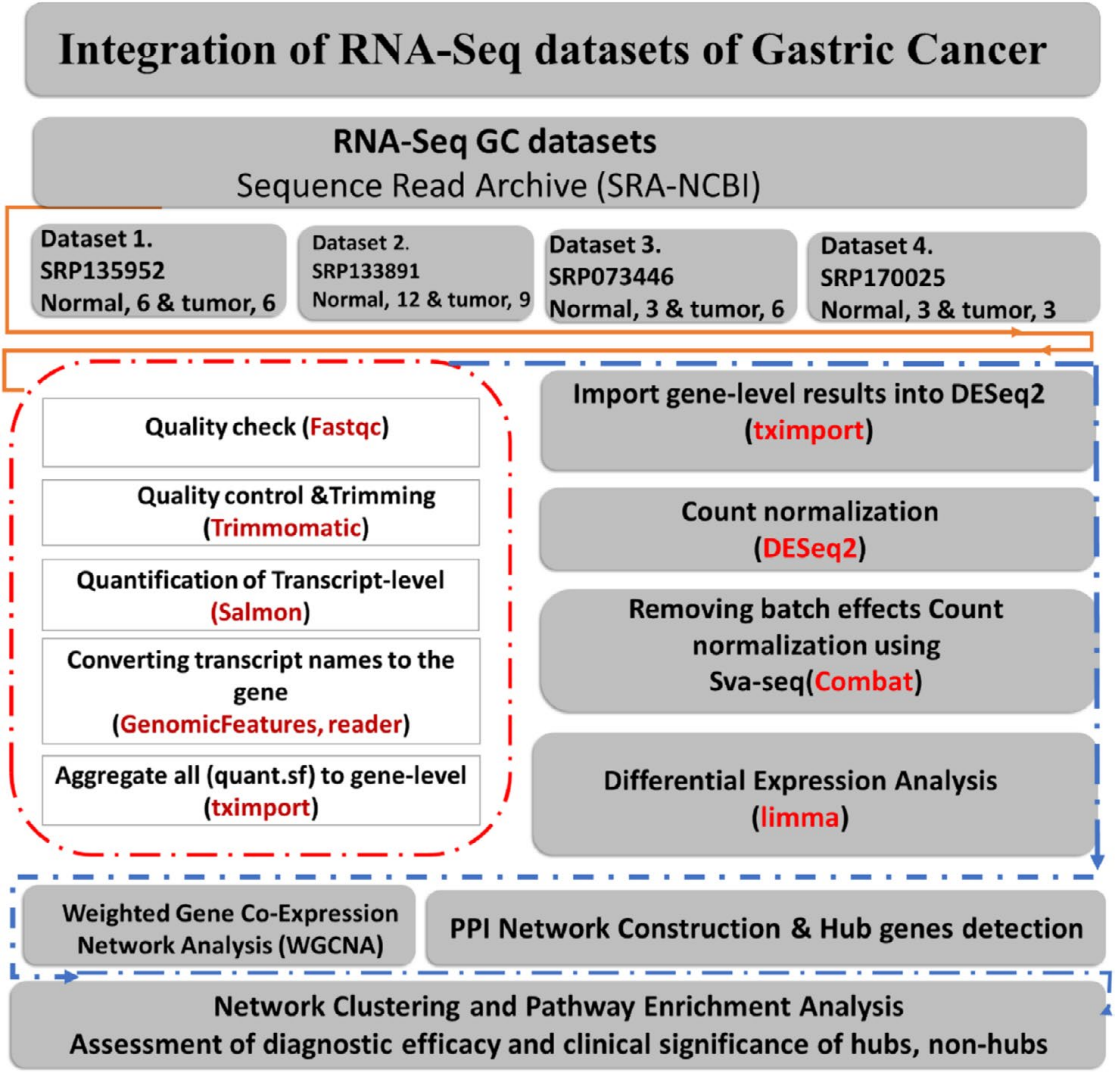
The pipeline of integration of gastric cancer RNA‐Seq datasets. The orange solid lines, orange dashed lines, and blue solid lines represent the various steps of the study, specifically the collection of datasets, the analysis of Fastq datasets, and the interpretation of results using Cytoscape and the Enrichment database, respectively.

The workflow of the current study includes 15 steps presented in the pipeline (Figure 1). All the Fastq paired‐end of 48 mRNA sequencing profiles from GC were retrieved from the NCBI‐SRA using SRA‐Toolkit (https://github.com/ncbi/sra‐tools/wiki/02.‐Installing‐SRA‐Toolkit) (Table 1). In order to increase the accuracy of the integration results, only paired‐end GC datasets containing normal and tumor samples were selected.

**TABLE 1:** The RNA‐seq datasets of gastric cancer employed in the present study.

*R*	SRA study	Library layout	Experimental design (tumor/normal)	Organization name
1	SRP135952	Paired	12 (6/6)	East China Normal University
2	SRP133891	Paired	21 (12/9)	Hallym University Medical Center
3	SRP170025	Paired	6 (3/3)	Fudan University Shanghai Cancer Center
4	SRP073446	Paired	9 (3/6)	National Chung Cheng University

### 2.2 RNA‐Seq Data Analysis, Batch Effect Removal, and DGE Analysis

After the quality check of Fastq paired‐end reads, more than 30% of the reads by quality below Q20 and adapters (first 12 bp of the reads) were trimmed utilizing the final version of FastQC [23] and Trimmomatic [24]. Subsequently, transcript‐level quantifications for each GC‐RNA‐Seq dataset were accurately quantified from cleaned reads according to the annotation file (Gencode.v99) using Salmon [25].

After obtaining all quant files related to each dataset, the tximport, GenomicFeatures, and Reader packages were employed to import the transcript level to the gene‐level files [26]. SVA‐seq (ComBat function) and DESeq2 packages were run on merged normalized values to detect and remove batch effects, low‐expression genes, and other undesirable sources of variation in merged datasets [27, 28]. To characterize significant DEGs and normalized log expression values, Limma with an adjusted *p* value threshold (Benjamini–Hochberg) of 0.05 and a fold‐change limit of |log2FC| ≥ 1 were used [29].

### 2.3 WGCN Analysis and PPIN Construction

Following the DGE analysis, WGCN analysis was applied to DEGs to filter and screen the co‐expression genes related to GC. All interconnected nodes and their interactions were derived through the igraph package using a specific formula and set of criteria [30]. Subsequently, the edges of the WGC network were then converted into weights assigned to the nodes, resulting in a giant component of the weighted network. Following this, the weighted adjacency matrix of the giant component was converted to a symmetric matrix, which was then used to create a new adjacency matrix employing the topological overlapping measure (TOM) function available in the WGCNA R package [31].

A comprehensive matrix representing all SPs within the weighted network was developed for every pair of nodes, implementing Dijkstra's algorithm [32]. This resulting matrix (Dj) served as the basis for determining the distance value associated with each node in the network. The average SPs from all noncore genes to node *j* were computed to derive Dj for each node. Then, the average SPs were subtracted from the core genes to reach node *j*. This value was divided by the average SPs to node *j* in the entire network. This rating system implies how close each node is to the core nodes [33, 34].

$$\mathrm{Dj}=\frac{\frac{\sum i\notin c\;\mathrm{SP}\mathrm{ij}}{\mathrm{NC}}-\frac{\sum i\in c\;\mathrm{SP}\mathrm{ij}}{C}}{\frac{\sum i\;\mathrm{SP}\mathrm{ij}}{\mathrm{NC}+C}}$$

The STRING database [35] was recruited to build a PPIN based on five sources of evidence. The extracted interactions were imported into the R program to visualize the PPIN.

### 2.4 Investigation of the Correlation Between the Expression Level of Hubs, Nonhubs, and IGFBP2

To evaluate the clinical significance of the identified PPIN nodes (both hubs and nonhubs), in conjugation with IGFBP2, Pearson's correlation and the statistical relationship were calculated pairwise among these groups based on their expression levels, employing the corTest package.

### 2.5 Comparative Examination of IGFBP2 Expression Levels, Mutation Frequencies, and Patient Survival Outcomes Across Different Types of Cancer

The comparison of the expression levels of IGFBP2 across different types of cancer, highlighting instances of both downregulation and upregulation of this gene, was analyzed using TCGA data through the UALCAN database.

An analysis was conducted to compare patient survival rates at both up and down expression levels of IGFBP2, utilizing various survival metrics such as disease‐specific survival (DSS), progression‐free survival (PFS), disease‐free survival (DFS), and overall survival (OS). This analysis was based on the TCGA dataset obtained from CBioPortal and employed the Survminer R package along with Kaplan–Meier (kME) methods. Additionally, genetic alterations of IGFBP2 were assessed across different types of cancer via Oncoprint [36].

### 2.6 Functional and Pathway Enrichment Analysis

Functional pathway enrichment analysis for all the significant PPIN clustered nodes was conducted using g:Profiler (http://biit.cs.ut.ee/gprofiler/) and EnrichR (https://cran.r‐project.org/package=enrichR).

## 3 Results

### 3.1 RNA‐Seq Data Normalization, Batch Effect Removal, and DGE Analysis

**FIGURE 2:**
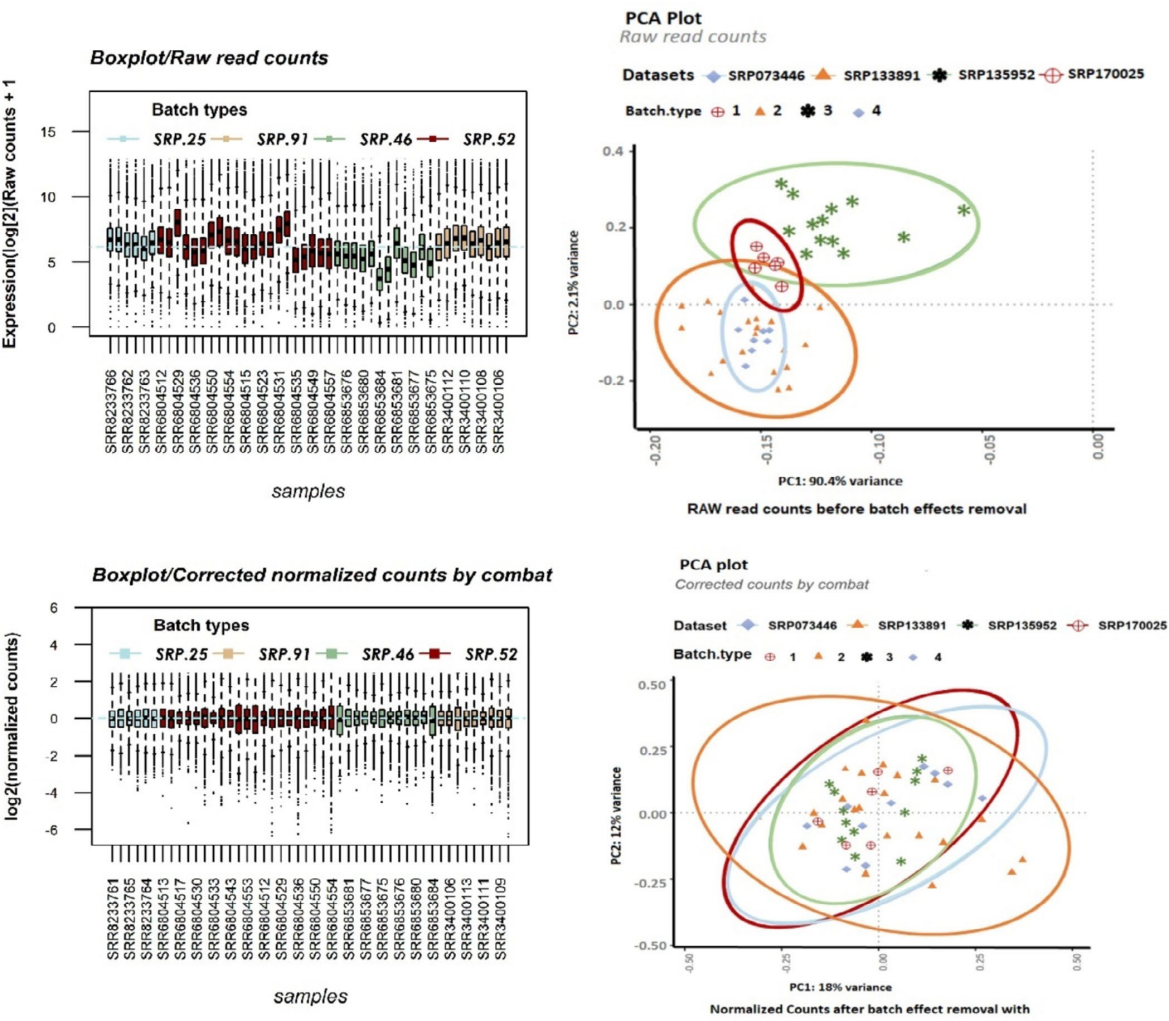
PCA plot and boxplot of the qualified samples based on Salmon's estimate before normalization and batch effect removal. Distribution of unnormalized counts (log2 (counts + 1)) per 48 samples (A) Normalized counts by combat (C) are displayed as boxplots and colored by dataset (batches). PCA plots show the first two components (PC1, PC2) of the principal component analysis, with percentages of variance associated with each axis. Colors identify datasets, and shapes identify batches. It can be seen from the PCA plot (B) that the first principal component shows a strong separation between the four batches. After applying ComBat to the counts (D), the differences between the four batches were largely reduced, and the percentage variation along PC1 and PC2 accounts for 17.9% and 12%, respectively, which is very small, and all symbol schemes (batches) remain the same place.

Following the integration of the four datasets, we performed clustering on the raw read counts (Figure 2B) as well as on the counts' postbatch effect removal (generated by ComBat) (Figure 2D) by principal component analysis (PCA) separately. The box plot illustrates the distribution of counts across samples for both unnormalized counts (Figure 2A) and batch‐corrected counts (Figure 2C). When normalization was performed separately for each batch followed by ComBat application, the optimal outcomes were achieved regarding batch similarity and the creation of a distinct normalized expression matrix suitable for DGE analysis.

After the normalization process and batch effect removal, a unique and normalized matrix with all samples (48 samples) was created and prepared to screen the DEGs between the GC and the control samples through the limma package. A total of 1000 DEGs (padj < 0.05) were obtained, of which 439 genes (padj < 0.05, log2FC ≥ 1) were significantly upregulated (uDEGs), and 133 genes (padj < 0.05, log2FC ≤ −1) were significantly downregulated (dDEGs) (Table S1).

### 3.2 Discovery of Hubs and Nonhubs From the WGC and Clustered PPI Networks

The SP of the WGCN analysis‐based scoring system explained in the material and methods section was used to detect nodes with shorter distances from the core genes (nodes with a positive *D*‐score), thereby extracting a smaller network from the huge component network. This network consists of 83 nodes containing 19 core nodes and 64 neighbors. The giant component of this network (core network) includes 16 seed nodes and 61 neighboring nodes. The diameter of the WGCN was six genes containing LIFR, LIF, PDGFR, COL1A2, FN1, CEACAM1, and RAB37. The network transitivity was about 50%, the edge density was about 14%, and the mean distance was 2.54 (Table S2).

**FIGURE 3:**
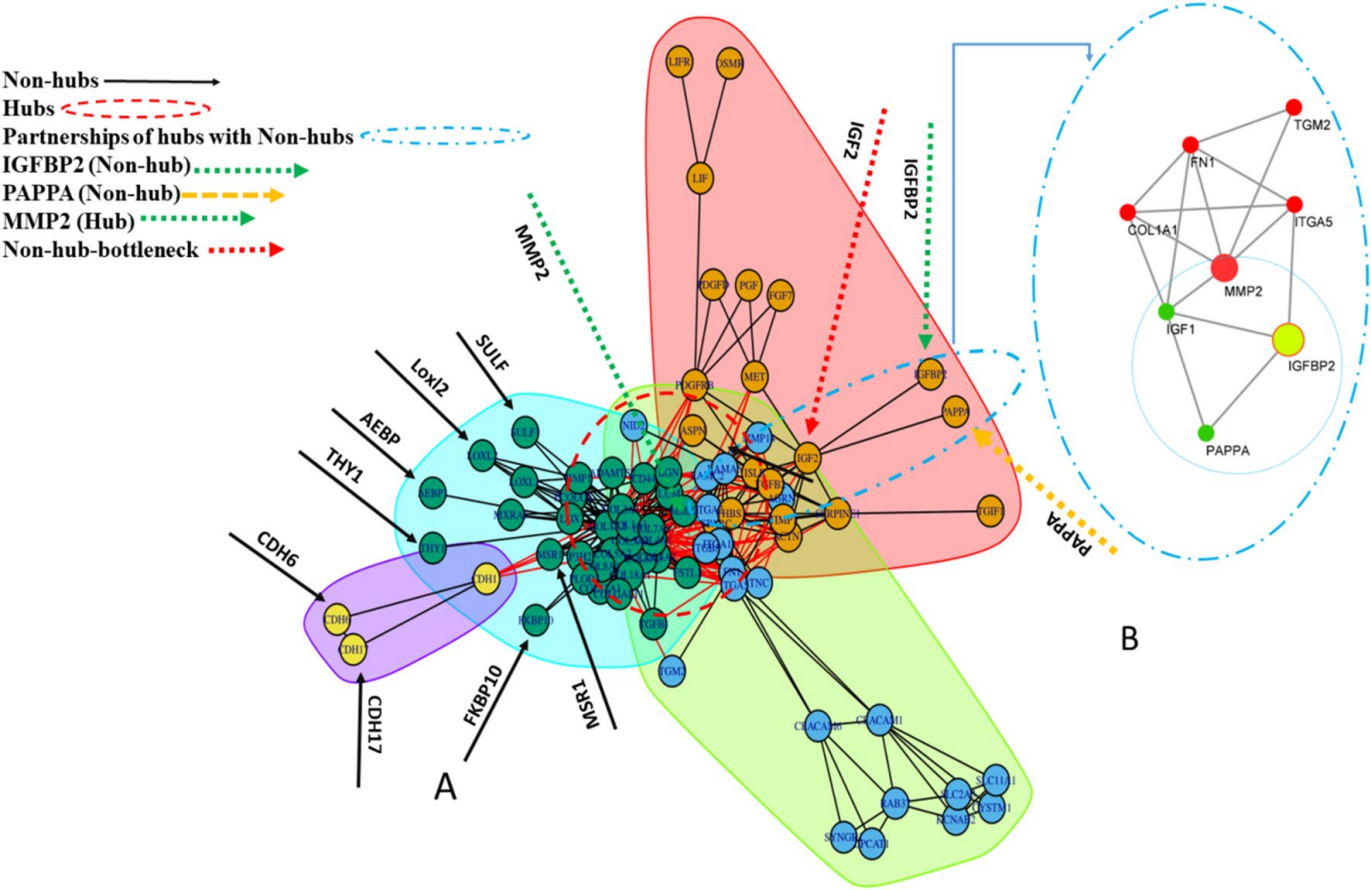
The clustering results of the PPIN. Four clusters are shown in the scheme, including Cluster A (dark green nodes), Cluster B (brown nodes), Cluster C (blue nodes), and Cluster D (yellow nodes). The nonhubs marked by the black arrows and the region by circular dashed red lines were hub nodes. The relationship between IGFFBP2 and nonhubs (PAPPA, IGF1) and hubs (MMP2, Col1A1, FN1, TGM2, ITGA5) is shown as circular dashed light blue lines. Cluster A is the largest cluster with 17 genes and hosts the collagen family genes, a key component of the core network.

In general, 83 co‐expressed DEGs (padj < 0.05) extracted from WGCN were selected to construct the PPIN. The PPIN nodes with degree greater than 13 and BC greater than 63.94 were considered hubs, including COL1A1, COL1A2, COL3A1, COL5A1, COL4A1, FN1, COL5A2, MMP2, ITGA2, SPARC, THBS1, and ITGA5. The direct neighbors of the hubs with a degree less than three and a BC less than nine, as well as indirect neighbors with two edge distances of the hubs with a degree less than three and a BC equal to zero were considered nonhubs, including NID2, MXRA5, FKBP10, SULF1, THY1, AEBP1, TGM2, CDH17, CDH6, TGIF1, IGFBP2, and PAPPA (Table 2). The clustering of PPIN was performed via a greedy optimization algorithm, resulting in four clusters (Figure 3).

**TABLE 2:** Statistical information of significant nodes (hubs and nonhubs) represented in PPIN based on *K*, BC, CC, and distance.

	Gene symbol	Features	*K*	BC	CC	Distance
Cluster A	COL1A2*	UP	37	450.23	0.5984	1.65
	COL4A1*	UP	27	73.23	0.5205	1.9
	COL5A2*	UP	23	65.7	0.481	2.05
	COL3A1*	UP	32	213.01	0.539	1.83
	COL5A1*	UP	28	100.81	0.5241	1.88
	COL1A1*	UP	39	436.86	0.608	1.62
	MMP2*	UP	20	91.19	0.5067	1.95
	FKBP10	UP	2	0	0.3838	2.57
	THY1	Up	1	0	0.3762	2.62
	MXRA5	UP	3	0	0.3918	2.52
	SPARC*	UP	18	70.08	0.4935	2
Cluster B	THBS1*	UP	18	65.72	0.4935	2
	TGIF1	UP	1	0	0.2923	3.38
	TGFBI	UP	4	0	0.4368	2.26
	IGFBP2	Down	1	0	0.3115	3.17
	PAPPA	UP	1	0	0.3115	3.17
Cluster C	FN1*	UP	24	496.28	0.5507	1.79
	ITGA5*	UP	18	269.01	0.5135	1.92
	TGM2	UP	2	0.66	0.3878	2.55
	NID2	Up	3	1.39	0.3938	2.51
	CDH17	Up	2	0	0.2901	3.4
Cluster D	CDH6	Up	2	0	0.2901	3.4
	SULF1	UP	2	0	0.3838	2.57
	AEBP1	Up	1	0	0.3762	2.62
	TGM2	Up	2	0.66	0.3878	2.55

*Note:* Hub nodes exhibiting the highest values of *K*, BC, and CC, along with the lowest distance, are indicated with asterisks.

### 3.3 Assessment of Diagnostic Efficacy and Clinical Significance of Hubs and Nonhubs

**FIGURE 4:**
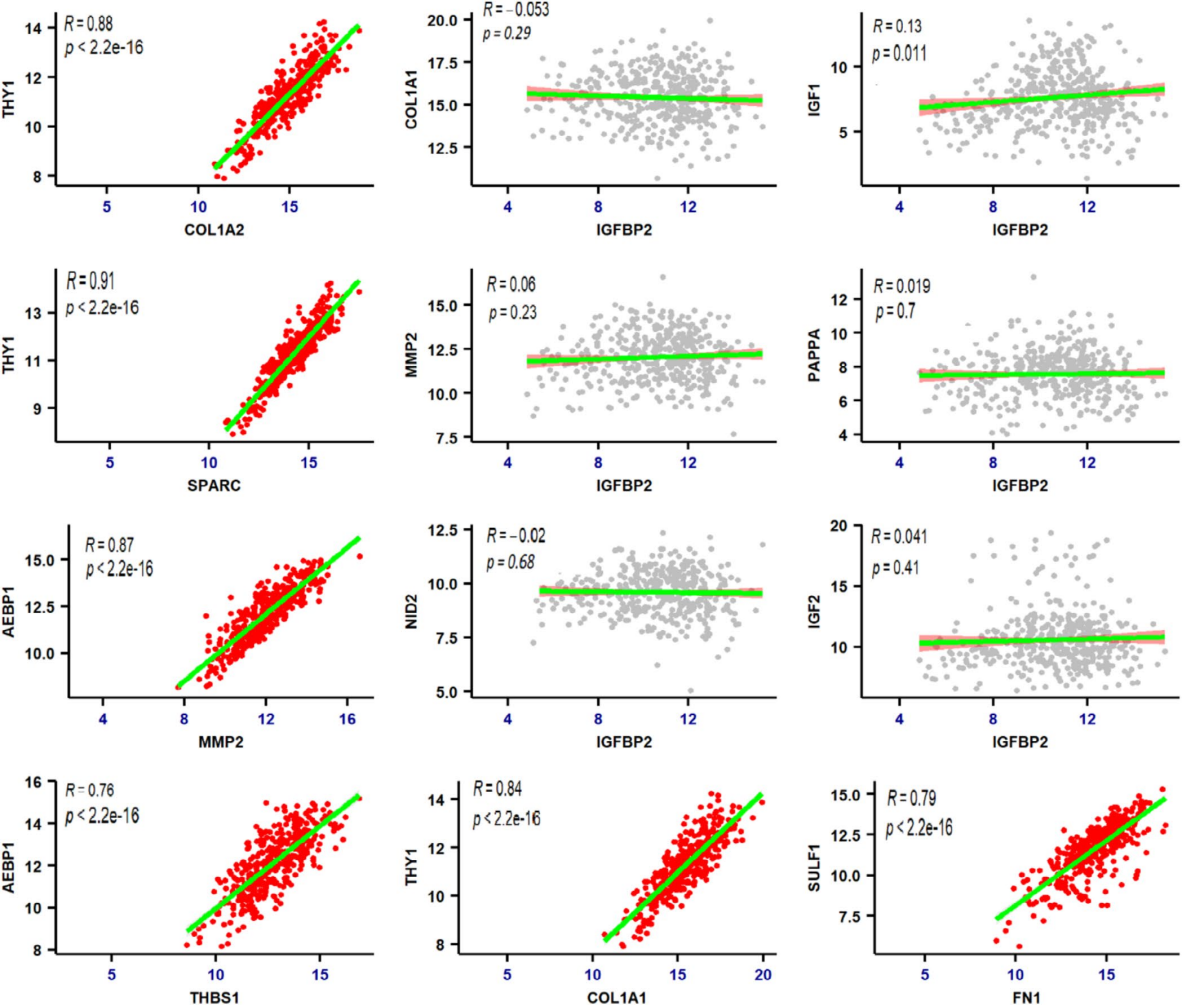
Pearson's correlation between expression levels based on the GC‐TCGA dataset. The correlation coefficients (Pearson) between the expression level of hubs, nonhubs, and IGFBP2 in GC samples (Nature TCGA, Pan‐Cancer Atlas, 293 patients). As shown, there is a statistically strong positive correlation between the expression level of hubs (MMP2, FN1, COL4A1, SPARC) and nonhubs (THY1, SULF1, AEBP1, NID2) (all plots with regression line colored green and dot (samples) colored red). The distribution of Pearson's correlation coefficients between the expression levels of hubs and nonhubs with IGFBP2 is shown as plots with gray dot plots. It is clear that the correlation between IGFBP2 and IGF1 was statistically positive, and contrary to the correlation analysis in our study, there is no significant relationship between hubs and nonhubs with IGFBP2.

In the current study, the nonhub nodes within each cluster exhibited significant upregulation, and since they have been proposed as potential biomarkers in numerous investigations, we speculated that these nodes may be associated with the hubs and could play a vital role in the progression of GC. To substantiate this hypothesis, we performed Pearson's correlation coefficient (*r*) test, which confirmed that all nonhubs correlate positively with the hubs. However, a statistically nonsignificant and negative correlation was observed between the expression levels of IGFBP2 and all hubs and nonhubs (Table S3). The results of this correlation analysis were derived consistent with our initial findings (Figure 4).

### 3.4 Examining the Expression Levels, the Mutation Percentage, and the Survival Outcomes Associated With IGFBP2 Across Various Cancer Types

Based on correlation analysis, the IGFBP2 gene, identified as a nonhub, exhibited significantly divergent trends compared to both hub and nonhub genes. Consequently, an independent assessment of this gene was conducted, focusing on comparative expression levels across various cancer types, mutation rate analysis, and multiple survival metrics, including OS, DSS, PFS, and DFS, to gain a deeper understanding of its characteristics.

**FIGURE 5:**
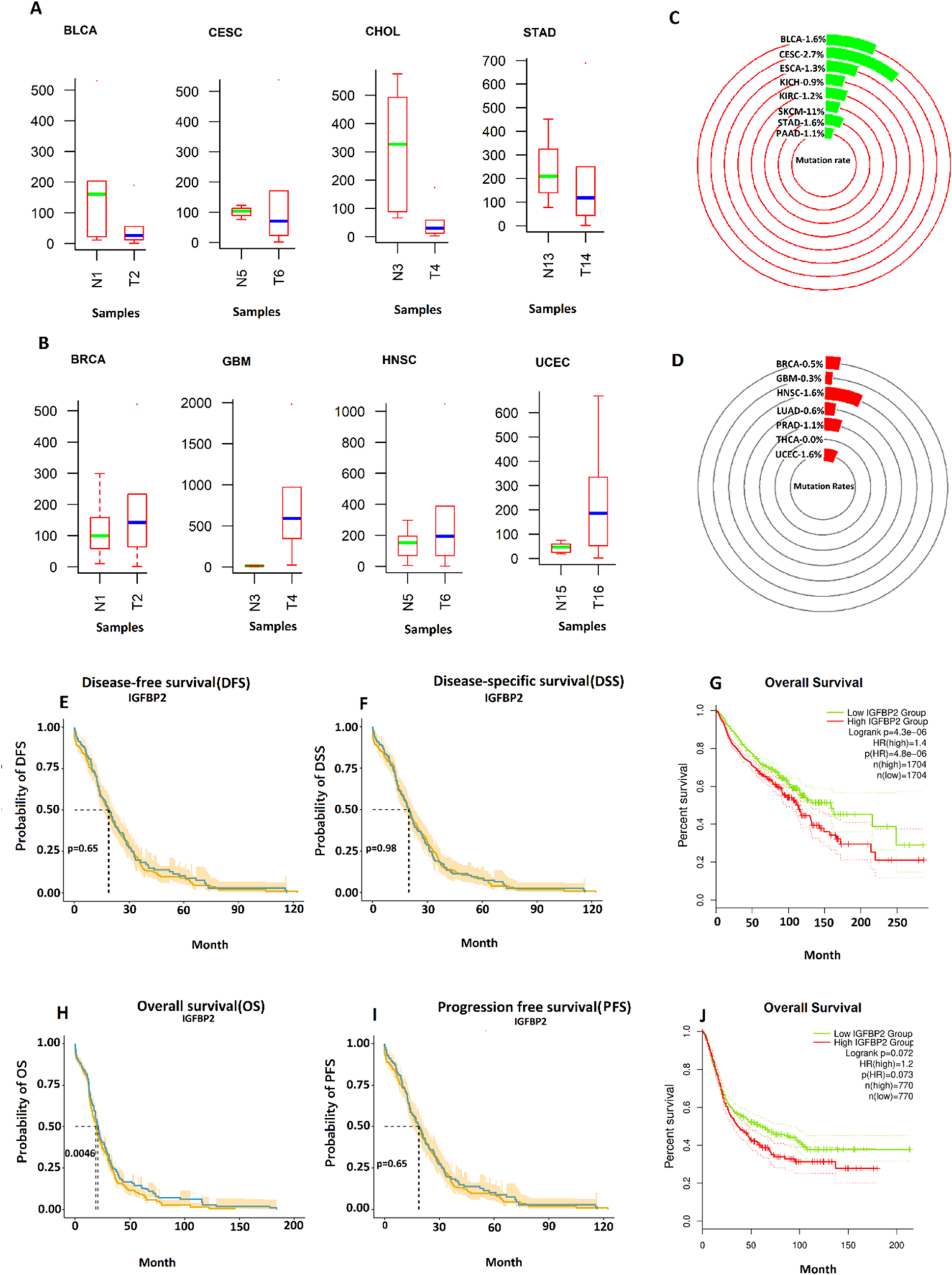
Prognostic value of IGFBP2: IGFBP2 mRNA expression was up‐ and downregulated in two states (normal samples [N], tumor samples [T]) in different cancer types (A, B). The *y*‐axis in (A, B) represents the expression level of IGFBP2 in different cancer types and the median of boxplots colored by solid red and blue lines. The genetic alterations of IGFBP2 in different cancer types (C, D) and overall survival (OS) with 95% confidence intervals (CIs) (dotted lines) of patients with GC (G, J) were shown. The prognostic impact of IGFBP2 on OS (H), DSS (F), PFS (I), and DFS (E) was evaluated. The y‐axis in (E, F) and (H, I) plots represents probabilities of DFS, DSS, DSS, OS, and PFS, respectively. In (E, F) and (H, I) plots, the green solid line represents the patient group with downregulated expression of IGFBP2, and the solid red line represents the patient group with upregulated expression of IGFBP2. OS analysis of GC patients with downregulation of IGFBP2 shows a significantly prolonged overall survival (*p* = 0.0046) in patients with GC.

As illustrated in Figure 5A,B, The expression level of IGFBP2 in numerous tumor samples is significantly downregulated, aligning with the findings of our study analysis (Table 2). This evidence further underscores the overall importance of our results.

The percentage of mutations in GC samples, including amplification, severe depletion, mRNA upregulation, and various mutations, was assessed using OncoPrint to ascertain whether the conflicting trend of this gene is associated with its mutation rate. In this context, the mutation rate of this gene was initially evaluated across all cancers exhibiting downregulation of this gene, including BLCA (1.6%) and CESC (2.7%), ESCA (1.3%), KICH (0.9%), KIRC (1.2%), SKCM (1%), STAD (1.6%), and PAAD (1.1%) (Figure 5C). Then, the same mutational analysis was performed for cancer types exhibiting upregulation of IGFBP2, including BRCA (0.5%), GBM (0.3%), HNSC (1.6%), LUAD (0.6%), PRAD (1.1%), THCA (0%), and UCEC (1.6%) (Figure 5D). In both cases, it was unexpectedly discovered that IGFBP2 displayed a mutation rate that was nearly identical with a minimal percentage of mutation in different types of cancers.

The findings illustrated in Figure 5G,H,J indicate that the outcome of the OS analysis, considering both the downregulation and upregulation of IGFBP2, reveals that downregulation of this gene is associated with more prolonged survival in GC patients. Similarly, as indicated in Figure 5E,F,I, the prognostic impact of IGFBP2 on DSS, DFS, and PFS shows that the downregulation of this gene is associated with prolonged survival.

The comparative analysis of gene expression alongside survival outcomes may validate the contradictory function of IGFBP2 in GC and other malignancies.

### 3.5 Functional and Pathway Enrichment Analysis

A noteworthy finding from the correlation analysis was the negative correlation of IGFBP2 with both hubs and nonhubs. To gain a deeper understanding of the relationship between these two groups and IGFBP2, a pathway analysis was conducted to identify the biological processes and signaling pathways in which IGFBP2 participates, apart from its association with hubs and nonhubs. The outcomes of the signaling pathway analysis indicated that multiple signaling pathways were significantly enriched, demonstrating the co‐presence of these groups in relation to IGFBP2, with correlations that were either positive or negative, as detailed in Table S3.

According to the results of both correlation and pathway analysis, as shown in Table 3, The main hubs (MMP2, COL1A1, FN1) and nonhubs (PAPPA, TGM2, TGFB1, IGF1) jointly cooperate in vital biological pathways including regulation of transport and uptake of insulin‐like growth factor (IGF), Oncostatin M and TNF‐alpha effects on cytokine activity, cell motility, and apoptosis. Meanwhile, IGFBP2 is associated with both nonhubs and hubs, and it is found in the majority of pathways.

**TABLE 3:** Pathway analysis based on BioPlanet pathway maps.

Series	Description	Adj‐*p*	Hubs	Nonhubs
1	Beta‐1 integrin cell surface interactions	2.14E‐16	COL1A1; COL3A1; COL5A1; COL4A1; COL5A2; FN1; ITGA5; THBS1; LAMA5	TGM2
2	Beta‐3 integrin cell surface interactions	4.52E‐09	COL1A1; COL4A1; FN1; THBS1	THY1, TGFBI
3	NCAM1 interactions	2.943e‐9	COL1A1; COL3A1; COL1A2	AGRN
4	Syndecan 1 pathway	3.41E‐09	COL1A1; COL3A1; COL1A2	
5	Extracellular matrix organization	9.41E‐14	COL1A2; COL4A1; COL5A2; COL3A1; COL5A1; COL1A1; MMP2; SPARC; THBS1; FN1; ITGA5; MMP14	NID2, PCOLCE
6	TGF‐beta regulation of extracellular matrix	0.0234\3885	COL1A1; FN1; TGFBI	NID2, AEBP1
7	Alpha‐9 beta‐1 integrin pathway	0.020489945	FN1	TGM2
8	Interleukin‐4 signaling pathway	2.89E‐04	COL1A1; COL1A2	THY1
9	Integrin beta‐2 pathway	0.025135122	TGFBI	THY1
10	Alpha‐M beta‐2 integrin signaling	0.046128092	MMP2	THY1
11	Signaling by PDGF	0.042372297	PDGFRB; COL1A1; COL3A1; COL1A2; COL4A2; COL5A1; COL4A1; PDGFD; COL5A2; COL6A3; THBS1	THY1
12	Oncostatin M	0.002014676	MMP2, COL1A1; FN1	IGFBP2, TGM2
13	Regulation of insulin‐like growth factor (IGF) transport and uptake by insulin‐like growth factor binding proteins (IGFBPs)	0.020360665	MMP2, FN1	IGFBP2, PAPPA
14	Diabetes pathways	0.025436156	MMP2	IGFBP2, PAPPA
15	Insulin‐like growth factor (IGF) activity regulation by insulin‐like growth factor binding proteins (IGFBPs)	9.64E‐05	MMP2	IGFBP2, PAPPA, IGF1

*Note:* Significantly enriched pathway terms for hubs, nonhubs, and IGFBP2 are shown. The results of the presence of two nodes with IGFBP2 in the biological signaling pathways are consistent with the results of cluster analysis (Figure 3). As shown by the cluster analysis, IGFBP2 is clustered with PAPPA, MMP2, IGF1, TGM2, FN1, and COL1A1.

## 4 Discussion

In most biomarker‐based studies, hubs extracted from the PPIN are introduced as biomarkers and used in clinical trials following a series of statistically validated methodologies [1, 37, 38, 39, 40]. Surprisingly, the present investigation aimed to identify new biomarkers for GC by retrieving and clustering both hubs and nonhubs from the PPIN using stringent statistical techniques. The analysis revealed that specific subcluster hubs and nonhubs within each cluster exhibit interconnections regarding their prognostic and diagnostic capabilities in GC.

Given the implementation of previous scientific reports, the significant hub nodes within the PPIN, such as COL1A1, COL1A2, COL3A1, COL5A1, COL4A1, COL5A2, FN1, MMP2, ITGA2, SPARC, THBS1, and ITGA5, play a crucial role in promoting cell proliferation, resistance to chemotherapy, invasion, and migration of GC cells, as well as facilitating distant metastasis. These nodes warrant further statistical and experimental validation in the context of GC research [41, 42, 43, 44, 45, 46].

However, considering the significant validation tests related to nonhubs (nodes with lower *K*, BC), including NID2, MXRA5, FKBP10, SULF1, THY1, AEBP1, TGM2, CDH17, CDH6, TGIF1, IGFBP2, and PAPPA, we hypothesize that these nodes can be associated with hubs and have the potential to be GC biomarkers.

An inconsistent trend of IGFBP2 was observed in both hubs and nonhubs. Therefore, various statistical validations were conducted to assess the specific potential of these two groups in relation to IGFBP2 and to determine whether the aforementioned elements are interconnected and possibly implicated in the GC signaling pathways.

Previous studies showed both types of nodes as validated biomarkers exhibiting upregulation in tumor conditions [47, 48, 49]. Overall, several nonhub proteins, including IGFBP2, PAPPA, CDH17, TGIF1, THY1, NID2, TGM2, and AEBP1, have been identified as oncogenic in specific stages of cancer [49, 50, 51]. Nevertheless, several contradictory studies (both in vitro and in vivo) suggested that these proteins may function as tumor suppressors [52, 53, 54]. Furthermore, several observations have illustrated the interaction between the identified nonhubs and hubs within our research [55, 56, 57].

Pearson's correlation analysis was conducted to validate the significance of relationships between the expression level of hubs and nonhubs. While, all nonhubs showed a strong, significant positive correlation with the hubs, a significant negative correlation was observed between the expression level of IGFBP2 and both hubs and nonhubs (Table S3). Pearson's correlation analysis conducted on TCGA data involving 293 patients with GC corroborated the findings of our study; however, the difference in IGFBP2 levels between the two groups of patients with GC was statistically insignificant. Given that IGFBP2 has not demonstrated a consistent trend in prior research or our current study, it was subjected to further examination through comparative analyses of various validation studies. The mutation frequency of this gene across multiple cancer types (1512 samples) revealed minimal variation in downregulated states (BLCA; 1.6%; KIRC; 1.2%; KICH; 0.9%; ESCA; 1.3%) and upregulated states (BRCA; 0.5%; GBM; 0.3%; PRAD; 1.1%; THCA; 0; LUAD; 0.6%). Consequently, it can be suggested that the fluctuating behavior of this gene in different cancers is not influenced by its mutation rate. As illustrated in Figure 5G,J, the low expression of this gene across assorted cancers, whether upregulated or downregulated, correlates with significantly longer survival. The alterations in the expression levels of this gene indicate an increase in various cancers such as BRCA, GBM, HNSC, LUAD, and UCEC, while a decrease is observed in other types, including BLCA, KIRC, KICH, and ESCA (Figure 5A,B). IGFBP2 is a member of a family comprising six insulin‐like growth factor binding proteins (IGFBPs). Current research indicates a varied expression pattern of this gene across different cancer types. For instance, studies have shown an upregulation of IGFBP2 in breast and cervical cancers [58, 59, 60], while downregulation has been noted in glioma, bladder cancer, and rhabdomyosarcoma (RMS) [61, 62, 63]. Consequently, it remains to be determined whether the overexpression or downregulation of this gene should be regarded as a potential biomarker. It raises the question of whether IGFBP2 functions as an oncogene or a tumor suppressor in cases of upregulation versus downregulation.

Considering the contradictory results, the multiple validation tests for IGFBP2, and the lack of a consistent trend, it seems that the best way to determine the exact role of this gene is to evaluate the interaction of this gene with hubs and nonhubs in the PPIN.

A selection of clustered genes suitable for investigation includes nonhub genes, such as PAPPA and IGF1, and hub genes, including MMP2, COL1A1, and FN1. Upon examining the interrelations among these genes, it became evident that they play a significant role in the regulation of IGF, as well as in the transport and uptake facilitated by IGFBPs and the regulation of IGFBP. Moreover, the activity of IGF acivity is regulated via the IGFBPs pathway.

Therefore, to clarify the function of this gene alongside its neighboring genes, a closer look at the IGF pathway should be taken. In addition to IGF‐I, IGF‐II, and IGFBP2, their receptors (IGF‐IRs) are components of the IGF pathway. Less is known about this pathway to date; however, the presence, interaction, and potency of IGFBPs with this pathway and both PAPPA and MMP2 could shed light on the complex role of this pathway in cancers [64, 65, 66, 67]. PAPPA (nonhub) and MMP2 (hub) have been introduced as cancer biomarkers.

Since high levels of IGFBPs can inhibit IGF‐stimulated functions by blocking binding to its receptors, the proteolysis of IGFBP2 is a pivotal mechanism in regulating and enhancing the carcinogenic effects of IGF‐signal transmission. A number of studies reported that PAPPA and MMP2 act as IGFBP2 proteinases, leading to cleavage of the IGF1/IGFBP2 complex. The breakdown of this system leads to the release of IGF1 from the complex, and this process (accumulation of IGF1) can facilitate cell migration toward the tumor [68, 69].

Given the role of IGFBP2 in the IGF system, it can be assumed that upregulation of this gene with blockade of the IGF pathway reduces the effect of tumorigenesis. On the other hand, the upregulation of PAPPA and MMP2 contributes to an increase in the accumulation of IGFBP2 in the cell through proteolysis of the IGF1/IGFBP2 complex, which in turn is involved in the stimulation of tumorigenesis by the IGF pathway. However, to justify the downregulation of this gene in our study and the TCG data, it can be proposed that the IGF pathway for the survival of its topicality also induces the expression of the hypoxia‐inducible factor 1 (HIF‐1), which can significantly reduce the expression of IGFBP2 [70, 71, 72, 73, 74].

From all the above interpretations, it can be concluded that the activity of IGFBP2 is dependent on the IGF pathway. Hence, upregulating the gene by blocking the IGF complex favors tumor suppression and is also a sign of activity accelerating proteolysis of two proteinase genes (PAPPA and MMP2). On the other hand, the downregulation of this gene may also be a biomarker of cancer recurrence since, in the absence of this gene, the IGF pathway will play a crucial role in tumorigenesis.

Given the statistically significant validation of nonhubs and hubs, it can be speculated that nonhubs and hubs have the potential to become GC biomarkers for the following reasons:

In light of the compelling evidence regarding IGFBP2 from both previous and current studies, we propose that IGFBP2, previously regarded as a dark horse, is influenced by the expression changes of nonhub genes (PAPPA, IGF1) and hub genes (COL1A1, FN1, MMP2) in GC. Significant downregulation and upregulation of this nonhub gene play a crucial role in tumorigenesis and tumor suppression. Alongside its neighboring genes within biological networks and signaling pathways, IGFBP2 plays a vital role in establishing a comprehensive signature throughout various stages of GC [75, 76, 77, 78, 79].

## 5 Conclusion

In summary, when presenting all the cumulative results of the study and considering the association of the IGFBP2 gene with hub and nonhub, it is essential to recognize that the designation of a gene as a biomarker is significantly influenced by its role within biological systems and gene networks. Specifically, nonhub genes within the PPIN exhibit lower *K* and BC values. However, it can be assumed that these elements appear to exert their effect on disease progression or suppression across the hubs. Therefore, additional computational, predictive modeling, and clinical research are required to determine which group, either hubs or nonhubs, exerts the most significant influence on the others and to assess the potential of nonhubs to serve as biomarkers. Undoubtedly, the advent of big data and machine learning present opportunities for understanding the interactions between hubs and nonhubs in tracking GC states, potentially leading to innovative solutions. Given that all selected datasets pertain to East Asia, we intend to investigate the relationship of this gene with its neighboring genes in the PPIN across various global populations. Furthermore, incorporating diverse variables such as age, gender, and climate into the DGE analysis enhances the precision of the research.

## Author Contributions

**Akram Siavoshi:** conceptualization, data curation, formal analysis, methodology, investigation, visualization, writing – original draft, writing – review and editing. **Mehran Piran:** data curation, formal analysis, software, methodology, investigation, validation, visualization. **Ali Sharifi‐Zarchi:** conceptualization, data curation, methodology, investigation, formal analysis, writing – review and editing. **Fatemeh Ataellahi:** writing – review and editing, methodology.

## Ethics Statement

The authors have nothing to report.

## Consent

The authors have nothing to report.

## Conflicts of Interest

The authors declare no conflicts of interest.

## Supporting information


**Table S1.** Differentially expressed genes (DEGs).

**Table S2.** The component of the WGC network.

**Table S3.** The correlation between expression level based on the employed GC datasets in the current study.

## Data Availability

The data that supports the findings of this study are available in the Supporting Information of this article.
